# Predicting Mechanical Properties of Cold-Rolled Steel Strips Using Micro-Magnetic NDT Technologies

**DOI:** 10.3390/ma15062151

**Published:** 2022-03-15

**Authors:** Hongwei Sheng, Ping Wang, Chenglong Tang

**Affiliations:** 1 College of Automation Engineering, Nanjing University of Aeronautics and Astronautics, Nanjing 210016, China; zjwx521@sina.com; 2 Nondestructive Detection and Monitoring Technology for High-Speed Transportation Facilities, Key Laboratory of Ministry of Industry and Information Technology, Nanjing 210016, China; 3 Central Research Institute of Baosteel, Shanghai 201999, China; tangcl@baosteel.com

**Keywords:** micro-magnetic NDT, mechanical properties, cold-rolled steel strip, polynomial fitting, improved GRNN model

## Abstract

Multiple micro-magnetic non-destructive testing (NDT) technologies are suitable candidates for predicting the mechanical properties of cold-rolled steel strips. In this work, based on magnetic domain dynamics behavior and magnetization theory, the correlation between electromagnetic characteristics extracted by multiple micro-magnetic NDT technologies and the influence factors was investigated. It was found that temperature and tension can subsequently affect the electromagnetic parameters by altering the domain structure and domain walls’ motion properties. Pearson’s correlation coefficients were employed to reflect the dependence of micromagnetic characteristics on influencing factors. The lift-off was determined as the largest influence factor among influence factors. A pseudo-static detection was reached by polynomial fitting, which could eliminate the influence of lift-off on the detection results. The number of training models was optimized, and the detection accuracy was improved via the improved Generalized Regression Neural Network (GRNN) model, based on the Gaussian Mixture Clustering (GMC) algorithm.

## 1. Introduction

Currently, the mechanical properties of cold-rolled steel strips (yield strength (Rp0.2), tensile strength (Rm), yield elongation (A), etc.) are commonly obtained by destructive experiments. However, destructive experiments are generally time-consuming, labor-intensive, wasteful in cutting, and lacking in data. Furthermore, the manufacture of ferromagnetic materials is growing automated, and its quality evaluation should not be limited to the laboratory but should also be parallel or embedded in the production process [1]. Yet, on the production line, in order to form feedback with the rolling process and provide a comprehensive warranty for customers, it is necessary to obtain steel strip mechanical properties of “meter-level” and “full-length”. These goals can be achieved by the appropriate micro-magnetic NDT methods.

Micro-magnetic NDT technologies, used to characterize the micro-structure [2,3,4,5,6] and stress state of ferromagnetic materials [7,8,9,10,11,12], are based on different magnetization principles [1,2,3,13,14,15]. They were first proposed in 1955 and have attracted wide attention since [1]. Desvaux et al. [16] and O’Sullivan et al. [17] studied the influence of residual stress, the micro-structure, elasticity, and plastic deformation of ferromagnetic materials on the BN method. The results show that the BN can be used to characterize the grain size of ferromagnetic materials and is in good agreement with those measured by X-ray diffraction. Wang et al. studied the coupling relationship between BN signal and stress and developed the BN stress testing instrument [18,19,20]. Boller et al. [21] indicated that the peak value of IP is sensitive to the influence of stress. Grimberg et al. [22] designed a system for non-destructive testing using IP and found that the coercivity measured by the IP method increases with the increase in fatigue times. Ryu et al. [23] studied the application of the reversible permeability method to evaluate material life under the long-term influence of a high-temperature environment for 1Cr-1Mo-0.25 V material in the steam turbine, and they found that the reversible permeability peak–peak distance decreases with the extension of material life. Wang et al. studied the relationship between incremental permeability, eddy current impedance, and micro-structure, and they found a new incremental permeability characteristic value to represent the average grain size and lattice friction of the material. The yield strength of the steel was estimated by using the new characteristics, and approximate accurate results were obtained [24].

Under field conditions, influence factors, for instance, temperature, working stress, residual stress, elastoplastic deformation of the material itself [25,26], and even material micro-structure [2,3,4,5,6,14,15], are usually accompanied. Moreover, influence factors not only reduce the detection accuracy but are also difficult to separate from the detection target. Fortunately, with the development of intelligent algorithms, a major breakthrough has been made for the integrated application of multi-micro-magnetic NDT technology and parameter separation [27,28,29]. XiuCheng et al. integrated Barkhausen and tangential magnetic field methods to predict the surface hardness of a 12CrMoV Steel Plate. The average error of the BP prediction model was reduced from 3.7% to 3.5% after adding the characteristic values of the tangential magnetic field [30]. The Fraunhofer Institute in Germany proposed the micro-magnetic, multi-parameter, micro-structure and stress analysis (3MA) technology. As well as this, the Fraunhofer Institute, using a multiple regression model, established a testing system to test mechanical properties based on 3MA technology [1,31,32,33].

However, the influences of the testing environment have not been discussed, and the influence degree has not been explored. Furthermore, to eliminate the negative effects, influence factors are commonly regarded as input models, the same as electromagnetic characteristics [1]. In the meantime, the detection model corresponds to a specific application object and does not have direct portability. Therefore, a large number of models will be generated, which will affect the training efficiency.

In this research, influence factor (temperature, tension, lift-off) experiments are carried out, and Pearson’s correlation coefficients between influence factors and magnetic characteristics are calculated, intuitively demonstrating that lift-off has the greatest influence on detection accuracy. After regression of lift-off, a pseudo-static detection is reached, which could eliminate the influence of lift-off on the detection results. Multiple micro-magnetic technologies combined with an improved GRNN model, based on the GMC algorithm, are used to predict the mechanical properties of cold-rolled steel strips online and reduce the number of offline training models. The confidence interval of predictions can reach over 95% at the absolute error level of 10%.

## 2. Methods

### 2.1. Online Micro-Magnetic NDT

**Figure 1:**
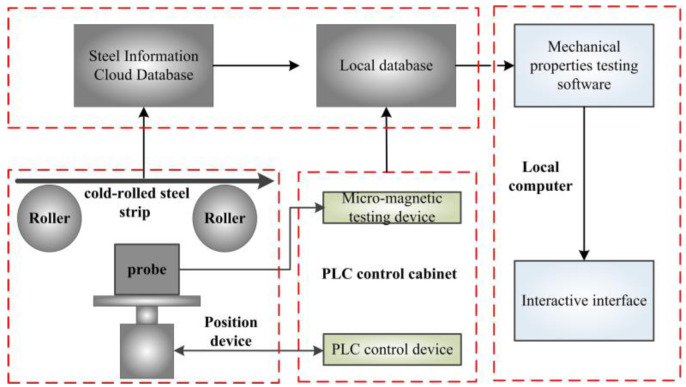
Online micro-magnetic testing system.

**Figure 2:**
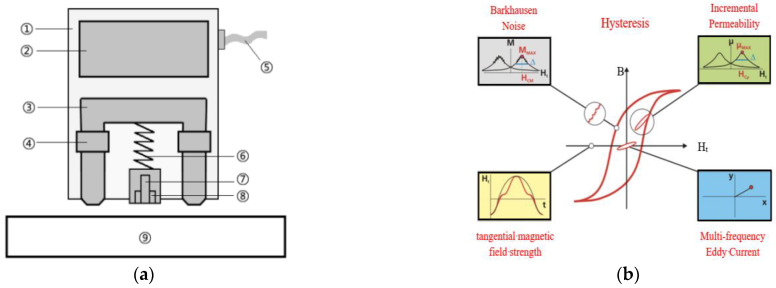
Probe (**a**) and testing techniques (**b**) [1]. Probe composition, ➀ protective case, ➁ probe electronic components, ➂ yoke, ➃ yoke coil, ➄ probe cable, ➅ spring-loaded sensor components, ➆ magnetic field sensor, ➇ inductive sensor, ➈ test sample.

As shown in Figure 1, the online micro-magnetic testing system for the mechanical properties of cold-rolled steel strips is divided into a hardware part and a software part, which is embedded after the hot dip galvanizing process of the cold-rolled steel strips. The workflow of the entire detection system and the role of its components can be explained as follows. Firstly, when the cold-rolled steel strips are running, rollers are used to minimize vibration and keep lift-off stable. Secondly, a probe is placed in a suitable position through a PLC control and position device, which can also send the signal of the lift-off between the probe and cold-rolled steel strips to a local database (LD). Thirdly, cold-rolled steel strips can be locally magnetized by the probe, which is a methodical combination of multiple micro-magnetic NDT techniques (TMF, BN, IP, EC) [1], as shown in Figure 2. Moreover, four kinds of original signals are picked up by ➆ and ➇, and processed by a micro-magnetic testing device. In the meantime, 41 electromagnetic characteristics, used to reflect the mechanical properties of cold-rolled steel strips, are stored in the LD. Fourthly, corresponding mechanical properties of cold-rolled steel strips are stored in the Steel Information Cloud Database (SICD). The LD obtains the corresponding mechanical properties in the SICD through Network Interface. All data (position signals, 41 electromagnetic characteristics, mechanical properties, etc.) are integrated into the LD, with the storage format as shown in Table 1. Finally, through Network Interface, integrated data are called by mechanical properties testing software (MPTS), which will automatically split data into training and validation sets and, then, built-in model training sets to target properties (Rm, Rp0.2, A).

The system is embedded in the cold-rolled steel strip production site, and in order to adapt to production, the test parameters are set as shown in Table 2.

### 2.2. Experiments

Usually, to compensate for test results, influence factors, considered as input characteristics similar to electromagnetic characteristics, participate in model training [1]. However, the influence factors have no direct relationship with the target properties (Rm, Rp0.2, A). Therefore, the scientific solution is to explore the internal relationship between the influence factors and electromagnetic characteristics based on the coupling mechanism and single variable experiment.

#### 2.2.1. Temperature Influence Experiment

The constant humidity and constant temperature test box is used to create an experimental environment with different temperatures. The controlled variable method is adopted in this experiment. During the experiment, the relative humidity is constant at 20%, the temperature range is 5–30 °C, and the step length is 5 °C.

In order to ensure that the temperature does not change during the detection, the probe and the sample are tied together and placed in a constant temperature and humidity box. The holding time under each temperature gradient is 30 min, and the micro-magnetic detection time is 1 min.

#### 2.2.2. Tension Influence Experiment

A hydraulic tensile testing machine is used to create different tensile test environments. The tensile testing experiment is carried out at room temperature (25 °C), and experiment parameters are set according to ATSM A370. The tension range is 5–75 MPa, and the step length is 10 MPa. Under each tensile gradient, the tension is retained for 30 min, and the micro-magnetic test is performed for 1 min.

#### 2.2.3. Lift-Off Influence Experiment

0.5 mm-thick Poly Vinyl Chloride (PVC) plats are used to control lift-off. The lift-off interval is from 0.5 mm to 7.5 mm, and the step length is 0.5 mm. Under each lift-off gradient, the micro-magnetic experiment time is 1 min. During the experiment, in order to avoid lift-off changes, an appropriate force is applied to the probe.

## 3. Influence Factors Analysis

### 3.1. Analysis of Experimental Results

**Figure 3:**
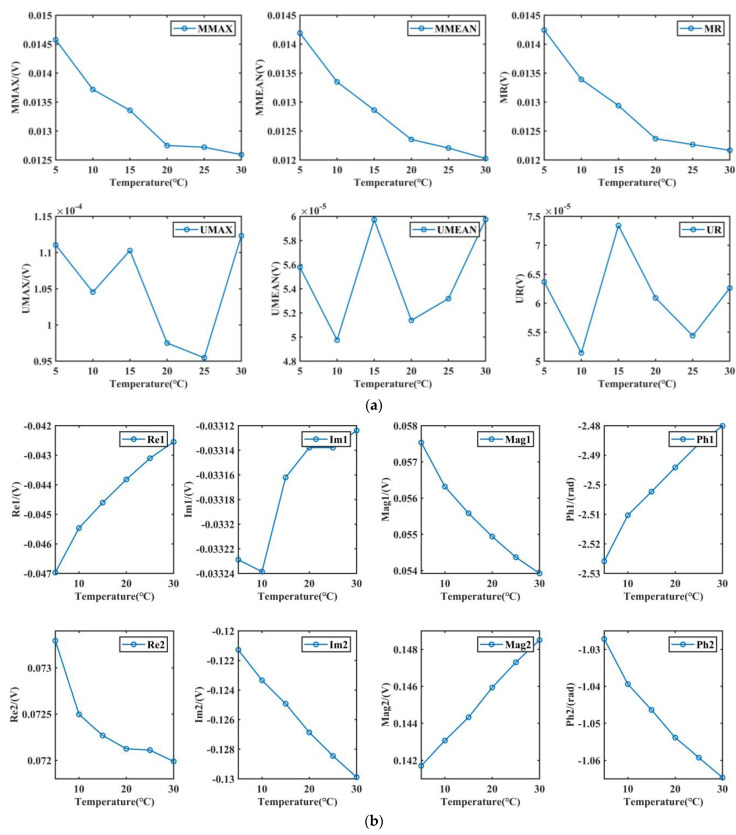

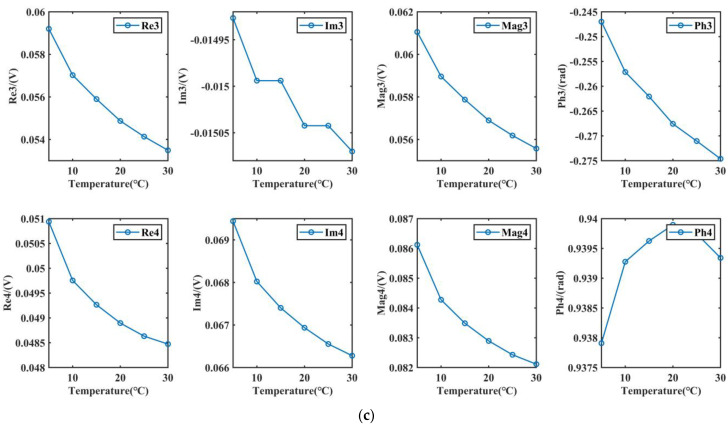
Characteristic signal changes under different temperatures. (**a**) Variation of BN and IP characteristics, (**b**) variation of EC real and imaginary, (**c**) variation of EC magnitude and phase.

Figure 3 illustrates the trend of electromagnetic characteristics with temperature. As the temperature increases, the BN characteristics (MMAX, MMEAN, MR) monotonically decrease, while the corresponding IP characteristics are irregular. The EC characteristics almost all show a monotonic change with temperature. The different responses to the temperature of the detection methods imply different physical principles.

The temperature rise will change the magnetostatic energy of the material and rearrange the magnetic domains, which could increase the number of magnetic domains. Meanwhile, the temperature rise will reduce the pinning effect threshold [34,35,36], thus reducing the energy consumption of the domain walls (DWs) breaking through the pinning. The main source of BN energy is the irreversible movement of domain walls (magnetic domain jump), which is closely related to the pinning effect threshold and the amount of pinning point. Therefore, on the premise that the material state does not change (assuming that the number of pinning points is unchanged), with the increase in temperature and under the same magnetization state, the energy consumption of the DWs’ breakthrough pinning effect decreases, which weakens the BN signal [37]. In addition, increasing temperature alters the energy distribution of the material, which makes the domains more chaotic [7]. The energy required to align the domain along the direction of the external magnetic field through the external magnetic field increases. Therefore, the irreversible magnetic moment deflection under the same external magnetic field, the higher the temperature, the more difficult it is to deflect, which also leads to the weaker BN signal. In other words, rising temperatures weaken the BN signal in at least two ways.

The IP characteristic signal corresponds to reversible magnetic domain movement, while the BN characteristic signal corresponds to irreversible magnetic domain movement. At low magnetic fields, reversible domain motion is caused by the expansion of domain walls, like elastic films, and by the shift of magnetic moments from the crystallography easy magnetization axis direction to the magnetization direction. With the cancellation of the external magnetic field, the domain structure will return to its original state. Compared with irreversible domain motion, reversible domain motion is more random under the action of temperature. The reasons for this phenomenon are explained as follows. The energy required to generate reversible domain motion is small, and to capture irreversible domain motion, IP requires an additional high-frequency excitation at the magnitude of mA [1,14,15]. Magnetostatics energy increases with increasing temperature [34,35,36]. In order to reduce the magnetostatic energy, the number of magnetic domains increases, which makes the domain sequence chaotic and the magnetocrystalline anisotropy energy increase [2,3]. In addition, with increasing temperature, IP characteristics present phase change, suggesting that the change of domain energy caused by temperatures is random. At low magnetic fields, magnetic energy is not guaranteed to overcome the temperature-induced energy change and make the magnetic moment rotate along the direction of magnetization. Therefore, the IP characteristics become unordered compared to the BN method.

Under the premise of fixed experimental conditions, the impedance of the eddy current coil is mainly affected by the electrical conductivity, effective permeability, and external excitation frequency. The increase in temperature will change the conductivity and effective permeability of ferromagnetic materials. In ferromagnetic materials, the higher the temperature is, the higher the hindrance effect of crystal defects on phonons is; this means that the free path of phonons decreases, which can decrease the thermal conductivity.

Meanwhile, according to Wiedemann–Franz law, which is defined as,

(1) $$\frac{\kappa }{\sigma T}=L_{0}$$

where *κ* is thermal conductivity, T is absolute temperature, σ is electrical conductivity, and $L_{0}$ is proportionality constant. The thermal conductivity of the material is proportional to the electrical conductivity. The increase in temperature reduces the thermal conductivity, as well as the electrical conductivity. According to the above analysis of IP, the material permeability is changed with the temperature increase. Under the same temperature, the effective permeability can be changed by different external magnetic fields [15]. The conductivity showed a monotonically decreasing trend with the increasing temperature. Temperature and excitation frequency increased, while the permeability changed in stages. Therefore, the nonlinear variation of multifrequency eddy current characteristics is mainly caused by permeability.

**Figure 4:**
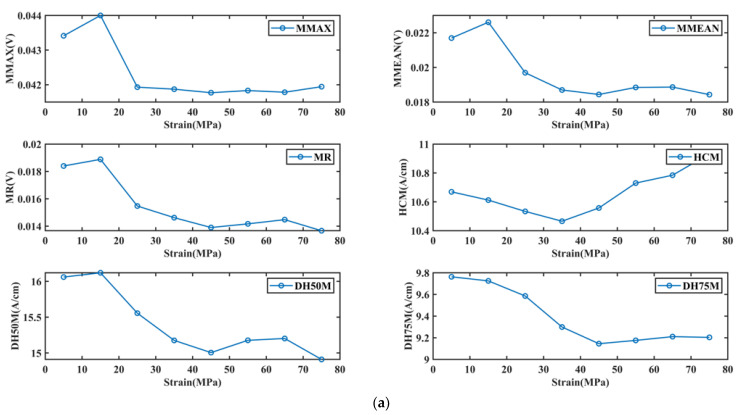

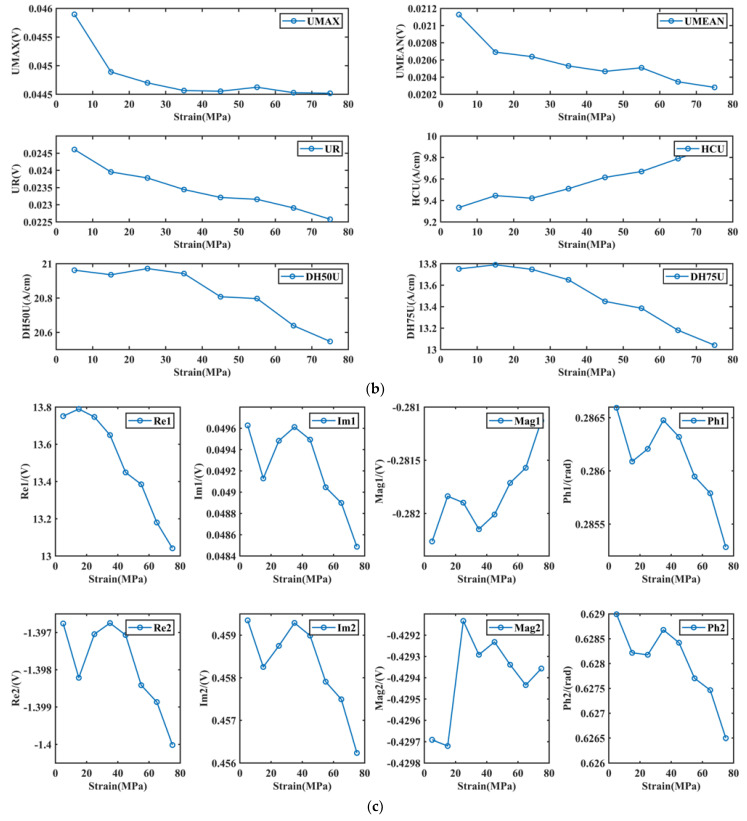

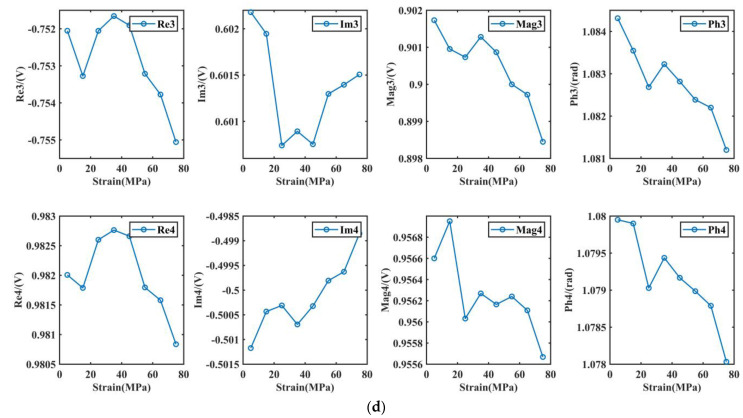
Characteristic signal changes with increasing external stress. (**a**) Variation of BN characteristics, (**b**) variation of BN characteristics, (**c**) variation of EC real and imaginary, (**d**) variation of EC magnitude and phase.

Tension is regarded as the external stress of the cold-rolled steel strips. When the strip is subjected to external stress, the internal stress of the strip will also change, which affects the magnetic domain movement during the magnetization process [7,8,9,10,11,12]. Figure 4 depicts the variation of some characteristics with the change of external stress. There is an inflection point for the BN and EC characteristics as the external stress increases. The IP characteristics almost all show a monotonically decreasing trend with increasing external stress.

External stress will change the internal stress state of the lattice and lead to an increase in dislocation density, which will increase the number of pinning points and hinder the DWs movement [38]. Therefore, BN can be affected by stress. When the direction of the magnetic field is perpendicular to the direction of the stress, with the increase in the stress (within the elastic strain range), DWs motion is given a new twist. At first, low stress with a gradient of 5 MPa is gradually applied, and the external stress interacts with stresses arising from dislocations, lattice defects, etc. within the crystal (the latter predominating). During this process, the ferrite does not undergo elastic deformation. Then, the increase in external stress changes the direction of residual stress in the lattice, and the principal stress direction is the external stress direction (external stress predominates) [25]. Ferrite deforms elastically, which increases dislocation density. At the same time, the free motion path of the domain perpendicular to the direction of principal stress becomes shorter [25], and the irreversible movement of the domain becomes more difficult, which weakens the BN signal. When the stress range is between 5 and 35 MPa, the external stress will rearrange the residual stress field in the lattice, which will cause the pinning effect of dislocation on the DWs to rise and fall, and then cause the phased change of the BN signal. When the stress exceeds 35 MPa, the external stress dominates the direction. With the increase in stress, ferrite deforms elastically in the direction of stress, which increases dislocation density in lattice and decreases BN energy. Whereas MMAX and MMEAN are less volatile, the monotonic increase in HCM and the decrease in HD50M and HD75M reduce the area of the butterfly curve. It is believed that the BN energy is decreasing with the increase in external stress. Between 5 and 35 MPa, the BN characteristics vary dramatically, while between 35 and 80 MPa, some BN characteristics are almost constant. This indicates that when the magnetic field direction is perpendicular to the external stress direction, the effect of intra-lattice stress on the BN is superior to that of the applied stress.

The IP characteristics (UMAX, UMEAN) change dramatically when the internal stress of the lattice is dominant but gently when the external stress is dominant. IP characteristics, such as DH50U and DH75U, have the opposite rule. UR and HCU vary almost linearly. In other words, when the internal stress of the lattice is dominant, external stress is applied to change the internal stress state of the material lattice and the magnetic axis. Therefore, under the action of the same magnetic field perpendicular to external stress, reversible expansion of magnetic domain and reversible deflection of the magnetic moment are more impeded, which will weaken the IP signal. The IP signal of the stress weakening in the crystal is reflected in the decrease in UMAX and UMEAN of the IP butterfly curve. When the external stress exceeds 35 MPa, the ferrite deforms elastically, and the magnetic domain changes to the external stress direction. At this point, a magnetic field perpendicular to the direction of the magnetic domain is applied, and the magnetic field overcomes the external stress to increase the energy consumption of the magnetic domain to the direction of the magnetic field (reversible motion). Therefore, under the same weak magnetic field, the larger the external stress is, the weaker the reversible magnetic domain is. The IP signal weakened by external stress is reflected in the decrease in the width of the butterfly curve (DH50U and DH75U).

Similar to the BN method, the EC also shows a phased change in the range of 5–35 MPa. This is because the external stress interacts with the internal stress of the lattice, causing electrical conductivity and effective permeability to respond differently to the result of their action. However, when it exceeds 35 MPa, the principal stress direction is external stress direction, and with the increase in pearlite elastic deformation, dislocation density increases, which increases the obstruction to phonons, so the thermal conductivity and electrical conductivity decrease. At the same time, the effective permeability is also decreasing. So, almost all EC characteristics show monotonicity variation within 35–75 MPa.

The cold-rolled steel strip is not completely smooth during the movement due to mechanical vibration; the distance between the bottom surface of the strip and the probe is variable. While the cold-rolled steel strip is magnetized, the skin effect exists, which is defined as,

(2) $$J=J_{S}exp\left(-\frac{x}{\delta }\right)$$

where *J_s_* is the current density on the surface of the cold-rolled steel strip, *x* is the distance between the current and the surface of the cold-rolled steel strip (lift-off), and *δ* is a coefficient related to the resistivity of the cold-rolled steel strip and the frequency of the alternating current, which is defined as,

(3) $$\delta =\sqrt{\frac{2\rho }{\omega \mu }}$$

where *ρ* is the resistivity of the cold-rolled steel strip, *ω* is the angular frequency of the alternating current, and *μ* is the absolute permeability of the cold-rolled steel strip. When the lift-off is changed, the magnetization state of the cold-rolled steel strip is changed due to the skin effect.

We can conclude that the detection results are a function of temperature, tension, and lift-off coupling effects in a constant magnetic field. The function between influence factors and electromagnetic properties is hard to explain by explicit relationships.

### 3.2. Analysis of Pearson’s Correlation Coefficients

**Figure 5:**
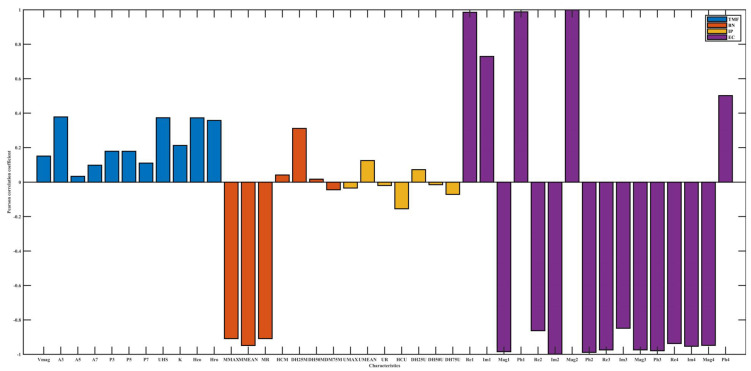
Pearson’s correlation coefficient between electromagnetic characteristics and temperature.

**Figure 6:**
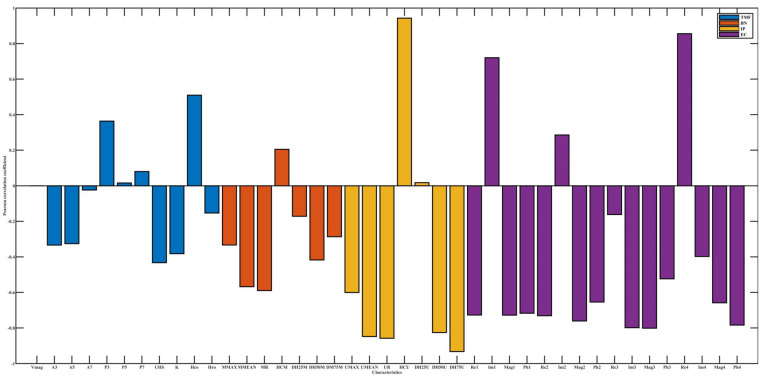
Pearson’s correlation coefficient between electromagnetic characteristics and tension.

**Figure 7:**
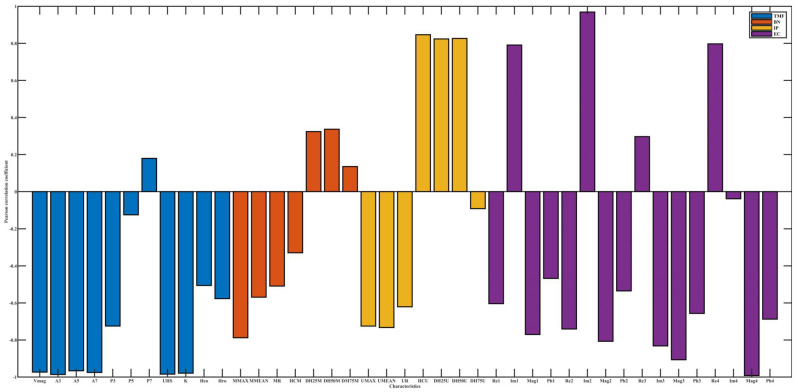
Pearson’s correlation coefficient between electromagnetic characteristics and lift-off.

Figure 5, Figure 6 and Figure 7, respectively, show the dependency of four micromagnetic methods to temperature, lift-off, and tension. Pearson’s correlation coefficients of TMF features for temperature and tension are almost all below 0.4, which indicates a weak correlation, while EC is highly correlated for almost all influencing factors. The micromagnetic NDTs have different degrees of dependence on influencing factors.

The Pearson’s correlation coefficient between IP characteristics and tension is significantly higher than that of temperature, indicating that temperature and tension can change the magnetization state of cold-rolled steel strips to different degrees.

The high Pearson’s correlation coefficients of both EC and influencing factors do not indicate that EC is not applicable to the online performance prediction of cold-rolled steel strips. Rather, it represents that EC is sensitive to changes in domain structure and domain motion, which are significantly influenced by temperature and tension.

The Pearson’s correlation coefficient signs between some characteristics and the influencing factors may be opposite, for example, the correlation coefficient of Re1 is opposite to that of temperature and tension, indicating that the influencing factors affect the magnetization state of materials from different mechanisms. Temperature can change the internal stress state to lower the domain pinning threshold. In the process of increasing tension to yield limit, the principal direction of stress and dislocation density will be changed, and the domain motion will be affected.

The values of Pearson’s correlation coefficient and its sign emphasize that the prediction of the mechanical properties of cold-rolled steel strips based on multiple micro-magnetic NDT technologies should consider the influence factors. From the degree of influence of influencing factors on electromagnetic characteristics, the Pearson’s correlation coefficient values between all the 41 micro-magnetic parameters and lift-off are almost all higher than 0.5. In other words, among various influencing factors, lift-off has the greatest influence on detection results.

**Figure 8:**
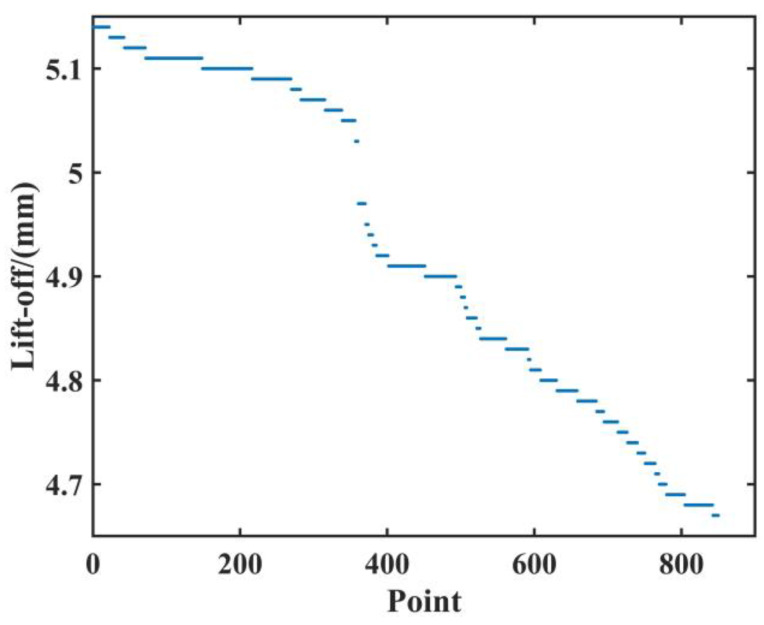
The changes of lift-off during steel coils operation.

In the process of field data investigation, we found that the tension and temperature of the same batch of steel coils are often altered within a tiny range. For example, the tension ranges from 23 MPa to 27 MPa, and the temperature of the cold-rolled steel strip is almost constant from quarter to quarter. Figure 8 shows the lift-off variation of approximately 100 coils of the same cold-rolled steel strip (each volume represented by 5–10 pieces of data). We can conclude that the fluctuation range is (4.67, 5.13) mm. Based on the results of the lift-off experiment, the electromagnetic features extracted would be affected. In order to obtain more accurate test results, it is necessary to compensate for the lift.

### 3.3. Electromagnetic Characteristics Regression Based on Lift-Off

**Figure 9:**
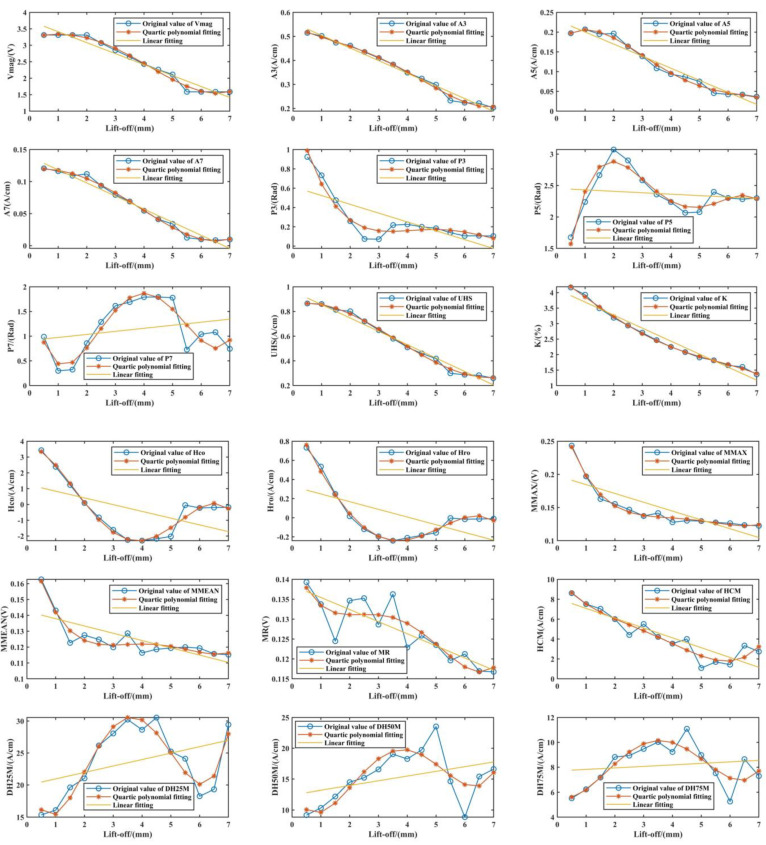
Polynomial fitting of some electromagnetic characteristics.

The least-squares method is a common method to deal with the curve-fitting problem, which is used in the regression of 41 electromagnetic parameters to achieve a pseudo static detection state. The results of linear fitting and quartic polynomial fitting are shown in Figure 9. The higher the Pearson’s correlation coefficient, the closer the result of the linear fitting is to the true distribution of electromagnetic characteristics. However, for the quartic polynomial fitting, it seems that it is not bound by Pearson’s correlation coefficient. In particular, when fitting moderate and low correlation characteristics (P5, P7, MR, DH25M, etc.), the results are closer to the real trend. Hence, the quartic polynomial fitting is emphasized in the lift-off regression.

## 4. Prediction Model and Results

Hundreds of products were produced by a cold-rolled steel strip production line, which would require hundreds of models to match with the steel strips. Hundreds of models could delay the online prediction efficiency, even causing a data lag, making them unable to satisfy the needs of online testing. It is necessary that a new method is employed to reduce the number of training models and accurately predict the mechanical properties of cold-rolled steel strips.

### 4.1. Improved GRNN Model Based on GMC Algorithm

The GRNN model was selected to predict ferromagnetic material mechanical properties in this study. First, GRNN is a kind of radial basis function neural network, which has excellent nonlinear approximation, high fault tolerance, and robustness, making it a suitable candidate for this study [39]. Second, GRNN does not require iterative training; the issue of local minima can be avoided [40], which reduces the possibility of getting a locally optimal solution.

GMC [41,42,43,44] based on the Expectation-Maximization (EM) cluster analysis algorithm is used to optimize the number of GRNN prediction models. GMC is a linear combination of multiple Gaussian distribution functions, which can fit any type of distribution, theoretically [45]. The EM algorithm is a maximum likelihood estimation method for solving problematic model parameters from incomplete data or data sets with data loss (with hidden variables), which is used to accelerate the convergence speed of GMC. The GMC based on EM mathematical algorithm is defined as

(4) $$\{\begin{array}{c} p\left(y|\theta \right)=\overset{K}{\underset{k=1}{\sum }}\alpha_{k}\emptyset (y|\theta_{k})\\ \emptyset \left(y|\theta_{k}\right)=\frac{1}{\sqrt{2\pi }\sigma_{k}}-\exp\left[-\frac{{\left(y-\mu_{k}\right)}^{2}}{2\sigma_{k}^{2}}\right]\\ L\left(\theta_{k}\right)=\overset{n}{\underset{i}{\prod }}p\left(x_{i};\theta_{k}\right),\theta_{k}\in \theta ,x_{i}\in R^{m},i=1,2,\cdots n \end{array}$$

where,$\;\alpha_{k}$ is coefficient, $\alpha_{k}\geq 0$, $\overset{K}{\underset{k=1}{\sum }}\alpha_{k}=1$, $\emptyset (y|\theta_{k})$ is Gaussian distribution density,$\;\theta_{k}=\left(\mu_{k},\sigma^{2}\right)$, $\;L\left(\theta_{k}\right)$ is likelihood function, *x_i_* is the *i* sample, *R^m^* is a sample set, and m is the dimension.

**Figure 10:**
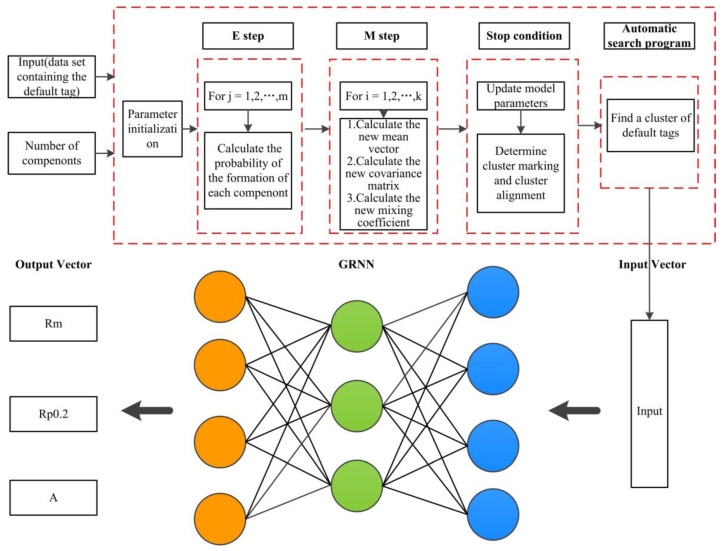
Model architecture.

As shown in Figure 10, a new method, an improved GRNN model based on the GMC algorithm, is proposed to predict mechanical properties. Mechanical properties (Rm, Rp0.2, A), obtained from the LD, are selected as input parameters of the GMC model.

### 4.2. Model Evaluation Functions

This study uses confidence interval (*CI*) and Root Mean Square Error (*RMSE*) as performance metrics to evaluate the prediction results. *CI* is an interval estimate. The value of *CI* close to 100% indicates the GRNN model prediction results have higher confidence. *CI* can be defined as

(5) $$CI_{10}=\frac{N_{10}}{N}\times 100\%$$

where *N* is the total number of data set, the number of testing samples whose average absolute value error (AAVE) is within 10% is expressed as *N*_10_. AAVE is the average value of the absolute value of the relative error. The value of AAVE close to 0 reflects the model has excellent prediction ability, defined as (6) and (7).

(6) $$E_{A}=\frac{\sum_{i=1}^{N}E_{i}}{yi}\times 100\%$$

where *E_i_* is the absolute value of the relative error, which can be defined as

(7) $$E_{i}=\left|\frac{y_{i}-y_{c,i}}{yi}\right|$$

where *y_i_* represents the mechanical performance value of the destruction experiment and *y_ci_* represents the predicted value of the mechanical performance of the non-destructive testing.

The value of *RMSE* is sensitive to individual extra-large or extra-small errors in a set of measurements. The estimated value obtained by the estimation method can minimize the root mean square error and, normally, does not generate individual large error values. Therefore, *RMSE* can better measure the measurement precision. *RMSE* can be expressed as

(8) $$RMSE=\sqrt{\frac{\sum_{i=1}^{N}(y_{i}^{\sim }-y_{i})}{N}}$$

### 4.3. Prediction Results

**Figure 11:**
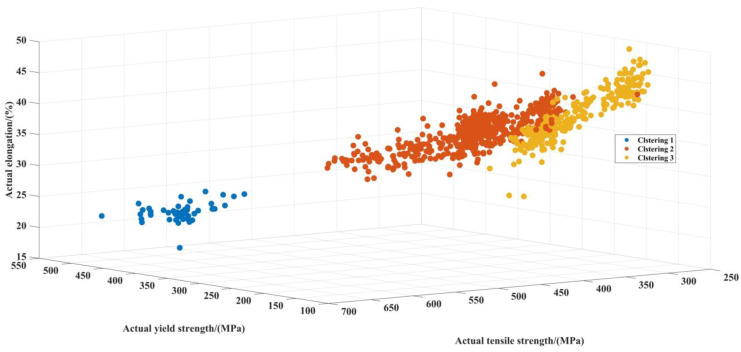
Clustering results of GMC.

In the process of cluster analysis, 24 kinds of cold-rolled steel strips, a total of 4800 sample data, are divided into 3 categories. Initially, the cold-rolled strip number 27,383, a total of 200 pieces, was marked. After cluster analysis, target data were divided into 3 clusters, a total of 1665 strips, which were composed of 13 kinds of cold-rolled steel strips. Cold-rolled strips with more than 150 pieces were removed, and 7 kinds of cold-rolled steel strips, a total of 1281 pieces, were retained after reprocessing. The results obtained by GMM is shown in Figure 11.

The model evaluation function is used to evaluate the results, which are shown in Table 3 andTable 4. At the absolute error level of 10%, after lift-off regression, the confidence interval of yield strength prediction is improved from 71% to 95% and the confidence interval of the tensile strength prediction increased from 94% to 100%. The confidence interval of the prediction of elongation at fault improved from 87% to 96%.

Concluding, the results demonstrate that the GRNN model exhibiting high accuracy and generalization ability can be adopted to predict mechanical properties. Hence, the new method could be used to reduce the number of training models, and through electromagnetic characteristics based on the new method and lift-off regression, the mechanical properties of the cold-rolled steel strips could be predicted without destructing the materials.

## 5. Conclusions

An online testing system was established, based on micro-magnetic NDT technology and an improved GRNN model, to predict the mechanical properties of cold-rolled steel strips. The following conclusions can be drawn:

1. Temperature and tension can subsequently affect the micro-magnetic characteristics by altering the domain structure and domain walls’ motion properties;
2. The lift-off was determined as the largest influence factor among influence factors, which could be eliminated by quartic polynomial fitting;
3. The number of training models were optimized by the improved GRNN model based on GMC, which could improve the detection accuracy;
4. The proposed system, embedded in the production process, will neither damage nor affect the usability of the steel strip and accurately obtain the steel strip’s mechanical properties.

## Figures and Tables

**Table 1:** Integrated data.

Strip Number	Tension (kN)	Lift-Off (mm)	Temperature (℃)	Micro-Electromagnetic Characteristics	Rm (MPa)	Rp0.2 (MPa)	A (%)
383	20	4.5	30	……	500	400	30

Note 1, Strip Number is the plate identification number. Note 2, Tension, acquired by the pressure sensor, is the driving force of the strip. Note 3, Temperature, obtained by a thermometer, is the detection temperature, usually room temperature. Note 4, All data in each row of the Table 1 is considered as one valid piece of data.

**Table 2:** Test standards.

Parameters	Value
Tension (kN)	20–40
Lift-off (mm)	4.5–5.5
Data Sampling Frequency of Micro-Magnetic Testing Device (Hz)	4
Temperature (℃)	20–30

**Table 3:** Prediction results without lift-off regression.

Mechanical Properties	Evaluation Function	
	$CI_{10}\;(100\%)$	*RMSE* (MPa)
Rp0.2	71	22.14
Rm	94	15.53
A	87	2.73

**Table 4:** Prediction results after lift-off regression.

Mechanical Properties	Evaluation Function	
	$CI_{10}\;(100\%)$	*RMSE* (MPa)
Rp0.2	95	8.58
Rm	1	5.98
A	96	1.76

## Data Availability

The datasets generated during and/or analyzed during the current study are available from the corresponding author on reasonable request.

## Nomenclature

**Symbol**	**Unit**	**Description**	**Testing Method**
A3, A5, A7	A/cm	Amplitude of 3rd, 5th, and/or 7th harmonics of Ht	Tangential magnetic field strength(TMF)
P3, P5, P7	Rad	Phase of 3rd, 5th, and/or 7th harmonics of Ht	
UHS	A/cm	Amplitude summation; A3 + A5 + A7 + A9	
K	%	Harmonic distortion	
Hco	A/cm	Coercive Field Strength, derived from TMF	
Hro	A/cm	Upper Harmonics of Ht at zero-crossing	
Vmag	V	Amplitude output voltage	
MMAX	V	Maximum of the M(H) curve within one cycle	Barkhausen noise(BN)
MMEAN	V	Time period average of the M(H) curve within one cycle	
MR	V	Measured value of M(H) at H = 0 A/cm	
HCM	A/cm	Coercive Field Strength, derived from the M(H) curve; H = HCM for M = MMAX	
DH25M DH50M DM75M	A/cm	Expansion of the M(H) curve at M = 0.25 MMAX, 0.50 MMAX, and 0.75 MMAX	
UMAX	V	Maximum of the u(H) curve within one cycle	Incremental permeability(IP)
UMEAN	V	Time period average of the U(H) curve within one cycle	
UR	V	Measured value of U(H) at H = 0 A/cm	
HCU	A/cm	Coercive Field Strength, derived from the U(H) curve; H = HCU for U = UMAX	
DH25U DH50U DM75U	A/cm	Expansion of the U(H) curve at U = 0.25 UMAX, 0.50 UMAX, and 0.75 UMAX	
Re1–Re4	V	Real part of the EC signal at Frequency No. 1, 2, 3, and 4	Multi-frequency Eddy Current (EC)
Im1–Im4	V	Imaginary part of the EC signal at Frequency No. 1, 2, 3, and 4	
Mag1–Mag4	V	Value of the EC signal at Frequency No. 1, 2, 3, and 4	
Ph1–Ph4	Rad	Phase of the EC signal at Frequency No. 1, 2, 3, and 4	
